# Typhoid Fever, Complicated with Epistaxis and Tetanus*Read before Buffalo Medical Club.

**Published:** 1880-11

**Authors:** Joseph Fowler


### Clinical Reports.

### TYPHOID FEVER, COMPLICATED WITH EPISTAXIS AND TETANUS.*

REPORTED BY JOSEPH FOWLER, M. D.

F. A., aged 22 years, called at my office late in the month of August, complaining of slight diarrhœa accompanied with headache and nausea.

The atmospheric conditions being favorable for the production of bowel troubles, I was inclined to consider his ailment of a transient nature, and prescribed simple remedies for his relief. He made a second visit two or three days later, and on the 29th day of September, I was sent for to see him at his home.

I found his condition apparently unchanged except less inclined to go about. He was not very ill and occupied his time lying down or setting up as his fancy took him.

I did not change my diagnosis, but watched his case suspiciously for a week, during which time there was an apparent improvement in his condition.

Monday, Sept. 6, I made my visit about 9 A. M. My patient was in bed with a temperature of 104°, pulse 100; intense headache and tongue brown, a little diarrhœa continuing, and had some epistaxis during the previous night. My diagnosis now was typhoid fever. Before leaving the house, I gave some instructions concerning local treatment, should the nose commence bleeding again. Soon after I had left, the epistaxis returned; they tried faithfully the suggestions I had made, but received no benefit. Becoming alarmed a messenger was despatched for me. Not being at home and waiting as long as they dare, Dr. Chas. A. Ring was called in, and remained with the patient till my return.

I reached the house 12.45 P. M. Dr. Ring had been there fully two hours, and had injected astringents into the nasal passages, and applied other local measures without abating the

* Read before Buffalo Medical Club.

hemorrhage. The bleeding had been so profuse that a bucket had been placed by the bedside, into which the blood was permitted to flow. To attempt to absorb it by means of cloths being held to the nostrils was like child's play. The only resource left was to plug the nares, which was done as soon as the proper conveniences could be made ready, for by this time the patient's lips had become palid, and the radial pulse was detected with difficulty.

The process of plugging I give in detail for reasons you will subsequently perceive. A new gum catheter was used, through which a soft cord had been drawn by means of the stilet. The catheter was then introduced into the bleeding passage, and as the extremity entered the throat, was seized with a pair of dressing forceps, and drawn into the mouth, the temperature of the body making it soft and pliable, and easily manipulated without endangering the soft parts to injury. The cord was made fast and the catheter withdrawn. A soft piece of sponge was attached to the string in such a manner that it would enter the posterior nares lengthwise, and not double up. The sponge was trimmed off, after it had been tied to the cord, so that it would make equal pressure upon the walls of the nares, thus lessening the danger of great pressure at one point, and none at another. The sponge was made smaller than is usual, and entered the cavity with great ease. This effectually controlled the hemorrhage, and the patient was placed upon the use of milk-punch freely. In the evening he appeared a little better, but was very weak. On the following morning at 9 o'clock, temperature 104°; pulse 104; respirations 28; his headache had ceased, and never returned again. He appeared much stronger and complained very little. On the next morning Sept. 8, 9:30 o'clock, temp. 102°; pulse 98; resp. 29. 6:30 P. M., temp. 102¾; pulse 100; resp. 24. His condition seemed to be improved in every respect.

Sept. 9, 9:30 A. M., temp. 101¼; pulse 90; resp. 24. 6:00 P. M., temp. 102½; pulse 98; resp. 25.

Sept. 10, 9:30 A. M., temperature 101 1/8; pulse 90; respiration 15. 6:10 P. M., temperature 102 3/4; pulse 92; respiration 25.

Sept. 11, 9:00 A. M., temperature 102 4/5; pulse 100; respiration 18. 8:20 P. M., temperature 102 3/4; pulse 100; respiration 25.

Sept. 12, 9:15 A. M., temperature 102 4/5; pulse 100; respiration 18. 8:30 P. M., temperature 102 3/4; pulse 100; respiration 25.

Sept. 13, 9:00 A. M., temperature 100 3/4; pulse 96; respiration 20. 6:10 P. M., temperature 103; pulse 102; respiration 23.

At my morning visit, on this date, the plug was removed, accompanied with some hemorrhage which ceased spontaneously; any effort to remove it prior to this, excited the bleeding. After its removal, as a precautionary measure, a clean, soft cotton cord coated with cosmoline was drawn in the nasal passage and permitted to remain. His temperature in the evening at 6 o'clock was 103°; pulse 102; respiration 23.

There was no diarrhœa, less tympanitis than before, and an improvement in his general aspect. Since the Monday before he had taken largely of milk-punch, and at times seemed to enjoy it.

The next day, Sept. 14, I left the city to be absent a couple of days, and left my patient to the care of Dr. Kilbourne. That morning he found a temperature of 101; pulse 100; respiration 22. At 6:00 P. M., it was 102; respiration 20; pulse 100. During the following night the doctor informed me that he was summoned in great haste and found the patient had well-marked lock-jaw. By making gentle traction on the cord which had been allowed to remain in the nasal passage since the removal of the plug, 36 hours before, caused an aggravation of the local trouble. The cord was at once removed and the nasal passage syringed out with an antiseptic solution.

Sept. 15, 11 A. M. temp. 99; pulse 96; resp. 20. In the afternoon the temp. was 102; pulse 100; resp. 22.

Upon my return on the morning of Sept. 16, I found his temp. 100 1/2; pulse 112; resp. 20. At 6:00 P. M., temp. 100 1/2; pulse 112; resp. 22.

Sept. 17, 7:30 A. M., temp. 100; pulse 110; resp. 23. 8:30 P. M., temp. 102¼; pulse 140; resp. 30.

Since Wednesday the patient could swallow but a small amount of fluid, and that with great difficulty. But to-day, Friday, Sept. 17, he was able to swallow more easily than for two days before. The muscles about the face were often seen to contract spasmodically, and the head was immovably fixed upon the shoulders, so intense was the muscular contraction. He craved food but could swallow only a little. The effort causing alarming strangulation.

At 12:35 P. M. his temperature was 103¾; pulse 141; respiration 36. His condition at this visit caused much anxiety for he had grown worse during the forenoon. He was able to breath freely through the affected nostril, although a small amount of clotted blood had occasionally been blown from it every day since the removal of the sponge plug.

I called again about 6 P. M., and just as I entered his room he asked for some milk, by means of gestures with which his attendants had become familiar. He was raised up in bed and a few drops of milk passed into his mouth with a spoon. After making several efforts he succeeded in swallowing it. A few drops more were again given him, when he was seized with a spasm and died in about three minutes. In this spasm it seemed that every muscle in his body was involved. The heart continued to beat for a minute or more after all other signs of life had gone. Efforts at artificial breathing were fruitless. On account of the extreme rarity of terminus following the simple and apparently harmless procedure of plugging the posterior nares, to which the result in this case seemed most probable to arise, I present you the details of the case.

